# Retraction: Complete Lumbar Spine Dislocation With Full Neurological Recovery

**DOI:** 10.7759/cureus.r75

**Published:** 2023-07-12

**Authors:** Abdulaziz M Alshamrani, Ahmed M Aldawsari, Saud A Alhassoun, Abdulrahman M Albahkali, Nawaf F Alhussain, Abdulaziz L Moqeem, Reem H Mohammed, Anhar M Hasanain, Afnan M Almutairi, Haider M Abu Shaheen, Abdullah A Al Qarni, Ali A Al Khalaf, Khalifah K Alfarhan, Fawaz M Alzubaidi, Malak Alshammari

**Affiliations:** 1 College of Medicine, Prince Sattam Bin Abdulaziz University, Al-Kharj, SAU; 2 College of Medicine, Imam Mohammad Ibn Saud Islamic University, Riyadh, SAU; 3 College of Medicine, King Saud Bin Abdulaziz University For Health Sciences, Riyadh, SAU; 4 Family Medicine, Directorate of Health Affairs, Jeddah, SAU; 5 College of Medicine, Ibnsina National College, Jeddah, SAU; 6 College of Medicine, King Abdulaziz University, Jeddah, SAU; 7 College of Medicine, Tabuk University, Tabuk, SAU; 8 College of Medicine, Imam Abdulrahman Bin Faisal University, Dammam, SAU; 9 College of Medicine, King Khalid University, Abha, SAU; 10 College of Medicine, King Faisal University, Hofuf, SAU; 11 College of Medicine, Umm Al Qura University, Mecca, SAU

This article has been retracted due to concerns regarding the provenance of this article as well as the credibility of the case reported therein. Repeated attempts to contact the authors and their institutions were unsuccessful, leaving the journal with no choice but to retract the article in order to correct the academic record.

